# Case Report: Early-Onset Charcot-Marie-Tooth 2N With Reversible White Matter Lesions Repeatedly Mimicked Stroke or Encephalitis

**DOI:** 10.3389/fped.2022.935721

**Published:** 2022-07-13

**Authors:** Huasheng Huang, Yu Zhang, Mingxiu Yang, Baorong Lian, Rui Guo, Liming Cao

**Affiliations:** ^1^Department of Neurology, Liuzhou People’s Hospital, Liuzhou, China; ^2^Nursing Department, Zhejiang Hospital, Hangzhou, China; ^3^Teaching Office, The First Affiliated Hospital of Shenzhen University, Shenzhen, China; ^4^Shantou University Medical College, Shantou University, Shantou, China; ^5^Radiology Department, Liuzhou People’s Hospital, Liuzhou, China; ^6^Department of Neurology, The First Affiliated Hospital of Shenzhen University, Shenzhen, China

**Keywords:** early-onset Charcot-Marie-Tooth disease type 2N, stroke-like onset, encephalitis, posterior reversible encephalopathy syndrome, case report

## Abstract

**Introduction:**

Charcot-Marie-Tooth (CMT) disease is a rare group of peripheral neuropathies with high clinical and genetic heterogeneity. CMT type 2N (CMT 2N) is a rare subtype of CMT with few clinical reports. The clinical presentation mimics that of other diseases, frequently leading to misdiagnoses. We present a case of CMT 2N with reversible white matter lesions (WMLs), which repeatedly mimicked stroke or encephalitis. We include a literature review to the improve management of this disease.

**Case Description:**

An 8-year-old boy was admitted to the hospital with slurred speech and limb weakness that had persisted for 1 day. Physical examination revealed lethargy, dysarthria, and a positive bilateral Babinski sign. Cerebrospinal fluid (CSF) analysis showed no abnormalities. Brain magnetic resonance imaging (MRI) revealed symmetrical abnormal signal areas in the paraventricular white matter and corpus callosum. The patient was suspected of having viral encephalitis and recovered rapidly after treatment.

He was hospitalized 3 years later for limb weakness, barylalia, and facial paralysis persisting for 1 day. MRI showed an abnormal signal in the bilateral corona radiata. He was suspected of having a stroke or encephalitis. He was completely recovered after treatment.

After a second 3-year span, he was admitted for another stroke-like episode. Physical examination revealed facial-lingual hemiparesis, mild atrophy of the left thenar muscle, decreased muscle strength in the extremities, and disappearance of the tendon reflex. MRI revealed more pronounced abnormal signals in the bilateral centrum semiovale and corpus callosum. Antibodies against autoimmune encephalitis were negative. A nerve conduction velocity (NCV) study showed motor and sensory four-limb nerve demyelination with axonal damage, most notably at the distal end. His symptoms were resolved after active treatment. A follow-up MRI showed the complete disappearance of the abnormal white matter signal. Whole exon sequencing showed a heterozygous mutation [c.2093C > T(p.Ser698Phe)] in the alanyl-tRNA synthetase 1 gene (*AARS1*). His mutation, clinical features, and electrophysiological testing led to a diagnosis of CMT 2N.

**Discussion:**

Early-Onset CMT 2N with reversible WMLs can often mimic stroke or encephalopathy. Affected individuals may show an atypical posterior reversible encephalopathy syndrome (PRES) on MRI. Careful family history assessment, physical examination, nerve conduction studies, MRIs, and genetic testing are essential for early diagnosis. Further studies are required to confirm these findings.

## Introduction

Charcot-Marie-Tooth (CMT) disease is a group of common peripheral neuropathies with high clinical and genetic heterogeneity (1). CMT has an overall prevalence of approximately 1/2,500 and is divided into two main subtypes, demyelinating (CMT 1) and axonal (CMT 2), based on neuroelectrophysiological findings (2, 3). The diagnosis of CMT 2 is based on autosomal dominant chronic axonal sensorimotor neuropathy with median motor nerve conduction velocity (NCV) (4, 5). CMT 2 accounts for approximately 30% of the overall cases of CMT, with a prevalence of approximately 1/10,000 (6).

Charcot-Marie-Tooth 2 is typically characterized by late age of onset, axonal damage, chronic progressive muscle weakness, atrophy of the distal extremities, and a decrease or loss of deep tendon reflexes, with or without mild sensory impairment (7). CMT subtype 2N (CMT 2N) is an autosomal dominant subtype caused by the alanyl-tRNA synthetase gene (*AARS*), part of the cytoplasmic aminoacyl-tRNA synthetase family (8). Mutations in this gene decrease the enzymatic activity of AARS (8). There are a few clinical reports of CMT 2N, and the phenotype mimics other diseases. We present a rare case of CMT 2N with reversible white matter lesions (WMLs), which repeatedly mimicked stroke or encephalitis. We include a literature review to improve understanding and clinical management of the disease.

## Case Description

An 8-year-old boy was admitted to our hospital in July 2015 with acute-onset slurred speech and limb weakness for 1 day. The patient’s medical record indicated that his uncle and mother had claw feet. The rest of his medical history was unremarkable. Physical examination on admission revealed normal body temperature and blood pressure (BP), lethargy, dysarthria, and a positive bilateral Babinski sign. On admission, a lumbar puncture showed an increased cerebrospinal fluid (CSF) pressure of 200 mm H_2_O with a normal white blood cell count and protein level. Anti-Mycoplasma pneumoniae immunoglobulin M (IgM) in serum was positive, while anti-Toxoplasma IgM, anti-cytomegalovirus IgM, anti-rubella virus IgM, anti-herpes virus type 2 IgM, anti-Legionella pneumophila IgM, anti-Rickettsia IgM, anti-Chlamydia pneumoniae IgM, anti-adenovirus IgM, anti-respiratory syncytial virus IgM, anti-influenza A virus IgM, anti-influenza B virus IgM, and anti-parainfluenza virus type 1 IgM in serum were all negative. Brain magnetic resonance imaging (MRI) showed symmetrical abnormal signal intensity involving paraventricular white matter and the splenium of the corpus callosum (without retained films). He was diagnosed with viral encephalitis at a local hospital and was administered intravenous (IV) mannitol and ribavirin. The patient recovered fully and was discharged 1 week after admission.

**FIGURE 1:**
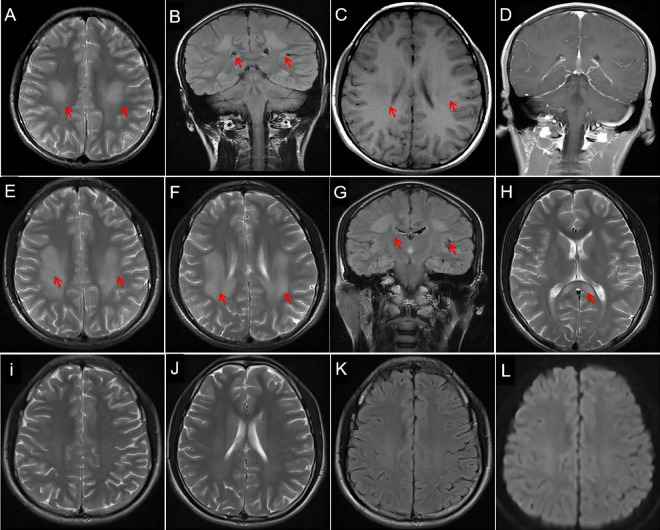
Evolution of lesions on brain magnetic resonance imaging (MRI). Hyperintensity lesions (arrows) in centrum semiovale and corona radiata on axial T2-weighted imaging (WI, **A**) and coronal T2-WI (**B**, arrows), hypointense lesions on T1-WI (**C**, arrows), and isointensity on contrast-enhanced MRI **(D)**. MRI reveals more hyperintensity signals in bilateral centrum semiovale (**E**, arrows), corona radiata (**F,G**, arrows), and corpus callosum (**H**, arrows) on T2-WI than before. Three months later, repeated MRI shows complete disappearance of the abnormal white matter signal on T2-WI **(I,J)**, fluid-attenuated inversion recovery imaging **(K)**, and diffusion WI **(L)**.

He was admitted a second time 3 years later in July 2018, where he presented with limb weakness, barylalia, and distortion of the commissure for 1 day. Physical examination revealed a BP of 116/82 mmHg, shortness of breath, left hypophysis, shallow nasolabial sulcus on the left side, distortion of the commissure, decreased muscle strength (4/5) in the four limbs, and a probable positive Babinski sign. Laboratory analysis showed that white blood cell count, liver, kidney, and coagulation function parameters were within normal limits. Blood electrolytes, hemoglobin, C-reactive protein, thyroid hormone, and myocardial enzyme levels were normal. Blood gas analysis, routine CSF analysis, CSF protein level, CSF culture, and sputum smear showed no significant abnormalities. Echocardiography, intracranial vascular ultrasonography, brain and chest computed tomography (CT), electroencephalography, and cervical and thoracic spine MRI showed no obvious abnormalities. Contrast-enhanced brain MRI demonstrated abnormal signals in the bilateral centrum semiovale and corona radiata (Figures 1A–D). His platelet count (323 × 109/L) was slightly increased. Stroke or encephalitis was suspected and treated with antiviral and symptomatic treatments to improve his cerebral metabolism; he recovered completely.

He presented with a 1-day history of perioral numbness on his third admission in April 2021. Physical examination revealed a BP of 126/79 mmHg, left peripheral facial paralysis, tongue deviation to the left, bilateral frontal and maxillary sinus tenderness, mild atrophy of the left thenar muscle, bilateral claw foot, decreased muscle strength in foot dorsiflexion (4/5), and no tendon reflex. Brain CT revealed suspected low-density foci in the corpus callosum. MRI revealed more obvious abnormal signals in the bilateral centrum semiovale (Figure 1E), corona radiata (Figures 1F,G), and corpus callosum (Figure 1H) when compared to his previous MRI. Brain magnetic resonance angiography showed no obvious abnormalities. Needle electromyography and NCV studies showed motor and sensory four-limb nerve demyelination with axonal damage, mainly at the distal end of the peripheral nerves [the median nerve, tibial nerve, and common peroneal nerve with bilateral reduced motor NCV (MNCV) and decreased amplitude; the ulnar nerve with bilateral reduced MNCV and decreased right amplitude; the median nerve, ulnar nerve, and superficial peroneal nerve with bilateral reduced sensory NCV and decreased amplitude, Table 1]. The CSF cell count was normal, but CSF protein levels were increased (480 mg/L, reference range: < 450 mg/L). CSF antibodies for autoimmune encephalitis were negative. He was treated with antibiotics, immunotherapy (IV dexamethasone), oral vitamins B1 and B12, and fluid replacement, and his symptoms were resolved. He was satisfied with the treatment he received and his recovery.

**TABLE 1:** Results of nerve conduction velocity studies in our patient.

		CMAP amplitude, mV, decreased percentage		MNCV, m/s, decreased percentage		SNAP amplitude, μ V, decreased percentage		SNCV, m/s, decreased percentage	
Nerve		L	R	L	R	L	R	L	R
Proband	Median	6.44	4.82↓, 19.7%	34.3↓, 31.4%	41.4↓, 17.2%	4.64↓, 53.6%	6.53↓, 34.7%	35.1↓, 29.8%	37.5↓, 25.0%
	Ulnar	7.79	4.56↓, 34.9%	36.8↓, 26.4%	36.2↓, 27.7%	7.14	4.90↓, 2.0%	36.5↓, 26.9%	34.8↓, 30.5%
	Tibial	0.87↓, 78.2%	3.04↓, 24.1%	31.1↓, 25.9%	31.1↓, 25.8%	NA	NA	NA	NA
	Sural	3.12	0.97↓, 67.7%	29.3↓, 30.2%	36.6↓, 12.9%	3.76↓, 24.8%	3.69↓, 26.1%	34.1↓, 18.8%	36.9↓, 12.2%
Proband’s mother	Median	3.55↓, 40.8%	2.10↓, 65.0%	45.3↓, 9.4%	35.3↓, 29.4%	3.04↓, 69.6%	2.22↓, 77.8%	35.6↓, 28.7%	40.0↓, 20.0%
	Ulnar	3.67↓, 47.6%	2.12↓, 69.7%	46.3↓, 7.4%	48.0↓,4.0%	3.87↓, 22.5%	3.59↓, 28.2%	35.7↓, 28.6%	38.5↓, 23.1%
	Tibial	0.20↓, 95.0%	0.90↓, 77.6%	34.6↓, 17.7%	30.9↓, 26.4%	NA	NA	NA	NA
	Sural	1.00↓, 66.6%	0.13↓, 95.7%	34.4↓, 18.0%	38.4↓, 8.6%	NA	NA	NA	NA

*MNCV, motor nerve conduction velocity; NA, not applicable; SNCV, sensory nerve conduction velocity; CMAP, compound motor action potential; SNAP, sensory nerve action potential; R, right; L, left; ↓, decline.*

**FIGURE 2:**
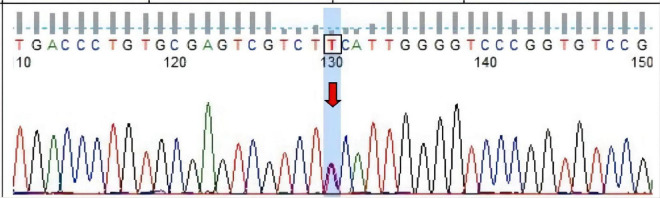
Sanger sequencing of heterotopic sites of *AARS1* in the proband. Forward sequencing shows the proband had [c.2093C > T, arrow, (p.Ser698Phe)] heterozygous mutation in exon 15 of *AARS1* (chr16).

Three months after discharge, MRI showed improvement in abnormal signal intensity in the bilateral basal ganglia, paraventricular white matter, and corpus callosum (Figures 1I–L). Brain perfusion imaging revealed no abnormalities. Whole exon sequencing (Figure 2 showed a heterozygous mutation in the alanyl-tRNA synthetase 1 (*AARS1*) gene [c.2093C > T(p.Ser698Phe)].

## Discussion

More than 20 different mutations can lead to CMT 2, most of which are autosomal dominant mutations, with a few with autosomal recessive inheritance (9). CMT 2 has significantly high genetic heterogeneity, and patients with different mutations may have similar clinical phenotypes. Therefore, it is difficult to use clinical symptoms and neuropathological diagnoses to distinguish the subtypes of CMT 2. More than 100 genes are associated with CMT 2, one of which was *AARS1* (10). *AARS1* mutations frequently result in autosomal dominant CMT 2N (11).

The largest CMT-related protein family is the *AARS*, which provides insight into the cellular pathophysiological mechanism, of which three are CMT 2-related (12). Research on the pathogenic mechanism of glyceryl-tRNA synthase (*GARS*) is more thorough than others. *GARS* mutations cause neurodegenerative diseases by interfering with the vascular endothelial growth factor/neuropilin-1 (VEGF/NRP1) signaling pathway, affecting protein conformation and opening new domains (13, 14). *AARS* mutations may have a pathogenic mechanism similar to *GARS*. To our knowledge, this heterozygous missense mutation [c.2093C > T (p.Ser698Phe)] in *AARS* has not been previously reported. We could not find this mutation in three population databases, the Human Gene Mutation Database, eso6500siv2_all, and 1000g2015aug. However, the mutation was included in the dbSNP147 database. Bioinformatics software predicted that this mutation is disease-causing. His mother had the same *AARS* mutation, and we concluded that his mutation was inherited from his mother.

### Clinical Features of Charcot-Marie-Tooth 2N

An increasing number of cases of CMT2 are identified in adulthood (15), especially in individuals older than 35 years (16, 17). Early-Onset cases, such as the present case, have rarely been reported. The typical clinical phenotype of CMT 2 includes foot deformity, areflexia, symmetricity, distal weakness, amyotrophy of the lower limbs, and hypoesthesia, which is more pronounced in the upper limbs than in the lower limbs, notwithstanding the genetic heterogeneity (18). Clinical features of CMT 2 usually include slowly progressive symmetrical muscle atrophy and weakness, predominantly in the distal limbs (19), which was also present in our case, but beyond sensory impairment. Brisk reflexes, extensor plantar responses, and asymmetrical muscle involvement can be considered parts of the CMT 2 phenotype (8). Compared to CMT1, patients with CMT 2 present with greater ankle weakness and increased and/or prolonged ankle dorsiflexion when standing (9). Signs and symptoms in our patient consistent with a distal peripheral neuropathy included distal muscle weakness and atrophies and depressed deep tendon reflexes, which were more pronounced in the lower limbs than in the upper limbs. Our patient developed progressive mild atrophy of the thenar muscle on the left hand and both lower extremities with the disappearance of tendon reflexes several years after the first admission. A long-term follow-up study revealed that patients with CMT 2 show a mild reduction in the strength of distal muscles of the upper limbs and proximal muscles of the lower limbs, worsening sensory function and muscle strength, a mild increase in walking disability (20), and an increase in disability (21).

Our patient also had symptoms of encephalopathy, such as facial and lingual paralysis and limb weakness. These symptoms are atypical clinical manifestations of CMT 2N, and we considered that they were related to reversible WML. We could not easily attribute the peripheral facial weakness in our patient to central WML because the upper facial muscles receive bilateral cortical innervation and the lower facial muscles are innervated by the contralateral cortex (22). However, our patient had bilateral symmetrical central lesions, which damaged the bilateral cortical innervation simultaneously, resulting in peripheral facial palsy and tongue deviation. In contrast, the upper facial muscles of some patients are mainly innervated by the contralateral cortex. When the contralateral cortex is damaged, similar peripheral facial paralysis occurs (23). Furthermore, a study showed that eye closure weakness was observed in 16 of 242 (6.6%) patients with unilateral stroke and central facial paralysis (24).

### Reversible White Matter Lesion or Atypical Posterior Reversible Encephalopathy Syndrome

To the best of our knowledge, stroke-like CMT 2N attacks with reversible WML have not been reported. X-linked CMT (CMT X1) with stroke-like episodes has been reported widely (25, 26), and prominent bilateral and symmetric restricted diffusion in the supratentorial white matter was thought to be characteristic of CMT X1-related stroke-like episodes (26). We learned from this case that CMT 2N can also mimic stroke and present similar imaging changes. The diagnostic criteria for PRES include neurological symptoms of acute attack, brain imaging abnormalities suggestive of vasogenic (focal) edema, and the reversibility of clinical and/or radiological findings (27). Neuroimaging reveals characteristic findings: symmetrical focal T2-hyperintensities mainly in the parietal and occipital lobes, followed by the frontal lobes and the cerebellum (28). However, atypical PRES (lesions involving only the subcortical white matter (29) and brainstem) has also been reported. Li et al. (30) reported two cases of PRES with bilateral, diffuse, but reversible deep periventricular WML without a clear posterior distribution, similar to the present case. MRI in our patient showed symmetric, reversible, bilateral, T2-weighted hyperintensities in the supratentorial white matter and corpus callosum, which are consistent with atypical PRES. We believe that although CMT 2N is not the cause of PRES, PRES onset may be related to CMT 2N. PRES-related focal neurologic deficits vary and correlate with the location of edema.

**Posterior reversible encephalopathy syndrome** (PRES) is associated with hypertension, renal failure, eclampsia, autoimmune conditions, and immunosuppression (28); however, our patient did not have any of these conditions. Mycoplasma pneumoniae infection (IgM-positive) was likely involved in the first episode. The pathophysiology of PRES may involve both brain hyper-perfusion due to increased BP and impaired permeability due to disruption of the blood-brain barrier (vasogenic edema) (31). Endothelial dysfunction is a key pathophysiological factor of PRES (32).

Our patient showed recurrent PRES. Previous studies have reported a recurrence rate of PRES of 3.8–5% (33, 34) and a higher recurrence rate (12.5%) among children (35). Recurrent PRES is mainly due to persistent increases in BP from various underlying diseases, autoimmune diseases, or treatment side effects (36). The commonest etiology of recurrent PRES is hypertension, which leads to vasogenic brain edema (35). Contrastingly, in the present case, no significant predisposing factor was identified before the second and third episodes (no hypertension, drug toxicity, or autoimmune disease). The patient’s normal BP during the three admissions did not mean that the patient’s BP 1 day before admission (immediately before the attack) was also normal. Several factors, such as strenuous exercise and tension, could cause an increase in BP. Therefore, we cannot rule out these factors, which can possibly increase BP and cause PRES. On the other hand, cerebral blood flow autoregulatory curves may also be shifted in special physiological states, making it a particularly vulnerable time for PRES to occur despite only moderate increments in BP (28).

### Electrophysiologic Studies

Electromyography and NCV in our patient showed motor and sensory four-limb nerve demyelination with axonal damage, most notably at the distal end of the peripheral nerve (Table 1). Interestingly, the NCV of the patient’s mother showed similar peripheral nerve damage (Table 1). Axonal patients are characterized by reduced compound action potential and a normal or mild reduction in NCV. NCV findings can help to determine the classification of CMT. CMT 1 indicates neuropathies in Schwann cells and peripheral nervous system myelinating glial cells, whereas CMT 2 indicates axonal neuropathies (37). According to the median MNCV, CMT is divided into demyelinating (CMT 1) with an MNCV below 38 m/s, axonal (CMT 2) with an MNCV above 38 m/s, and intermediate CMT (38).

### Diagnosis and Differential Diagnosis

This patient had been admitted multiple times due to a repeated presumed stroke or encephalitis. At that time, gene sequencing was not widely available, and a definitive diagnosis was difficult to make. Acute disseminated encephalomyelitis was considered, but the large-scale confluent WMLs were considered unusual, as would be the symmetry of this pattern. Multiple sclerosis, neuromyelitis optica, and myelin oligodendrocyte antibody spectrum disorders typically do not exhibit these patterns. Although there is a classic mimic of nearly all central demyelinating disorders, central nervous system lymphoma can be excluded based on symmetry, the good prognosis of patients, and lack of other findings.

### Treatment

Treatment of PRES typically focuses on managing the underlying cause and removing the offending agents. Immunosuppressive or cytotoxic medication is responsible for the neurological manifestations of PRES. Management of the underlying disease or precipitating factor leading to PRES is important (39). In addition, the management of hypertensive episodes and maintenance of normal BP are essential components of PRES treatment. Aggressive BP management in hypertension-induced PRES reduces associated morbidity (40). If possible, elimination of the triggering factor or management of the underlying cause should be initiated early during the course of the disease.

Our patient’s symptoms were improved after symptomatic treatment; however, he may have received some unnecessary treatment. There is no reliable and specific treatment for CMT 2N, and patients receive mainly symptomatic treatment. Nevertheless, studies on some promising treatments, such as valproic acid, are ongoing (12). Molecular mechanisms underlying CMT are diverse, and eliciting a specific therapy for each subtype will require much further study.

From our experience in the present case, we suggest that if an early diagnosis of CMT 2N can be made, unnecessary treatment can be avoided, and the progression of CMT 2N may be delayed. This case study expands our understanding of the clinical and imaging phenotype of CMT 2N. Further research is required to confirm these interesting findings and investigate the underlying mechanisms.

## Conclusion

We present a rare, heterozygous mutation of the *AARS1* gene found in a patient with early-onset CMT 2N with PRES, in which the clinical presentation mimicked stroke or encephalopathy. Careful collection and review of family history, physical examination, nerve conduction studies, brain MRI, and genetic testing are pivotal to the early diagnosis of CMT 2N. PRES may be an MRI feature of CMT 2N. Further studies are required to confirm these interesting findings and investigate the underlying mechanisms.

## Data Availability Statement

The original contributions presented in this study are included in the article/supplementary material, further inquiries can be directed to the corresponding author.

## Ethics Statement

The studies involving human participants were reviewed and approved by the Ethics Review Board of Liuzhou People’s Hospital approved this study design (No: 2022K Y-E-01). Written informed consent to participate in this study was provided by the participants’ legal guardian/next of kin. Written informed consent was obtained from the individual(s), and minor(s)’ legal guardian/next of kin, for the publication of any potentially identifiable images or data included in this article.

## Author Contributions

HH was responsible for the data collection, passing the ethical review, and writing the first draft. YZ revised and translated the manuscript. LC conceived the study and critically revised the manuscript. MY provided the constructive discussion. BL translated the manuscript. RG analyzed the imaging. All authors have read and approved the manuscript.

## Conflict of Interest

The authors declare that the research was conducted in the absence of any commercial or financial relationships that could be construed as a potential conflict of interest.

## Publisher’s Note

All claims expressed in this article are solely those of the authors and do not necessarily represent those of their affiliated organizations, or those of the publisher, the editors and the reviewers. Any product that may be evaluated in this article, or claim that may be made by its manufacturer, is not guaranteed or endorsed by the publisher.

## References

[1] Lupski JR Reid JG Gonzaga-Jauregui C Rio Deiros D Chen DC Nazareth L Whole-genome sequencing in a patient with Charcot-marie-tooth neuropathy. *N Engl J Med.* (2010) 362:1181–91. 10.1056/NEJMoa0908094 20220177 PMC4036802

[2] Luigetti M Fabrizi GM Taioli F Del Grande A Lo Monaco M. A novel LITAF/SIMPLE variant within a family with minimal demyelinating Charcot-marie-tooth disease. *Neurol Sci.* (2014) 35:2005–7. 10.1007/s10072-014-1833-2 24844793

[3] Saporta AS Sottile SL Miller LJ Feely SM Siskind CE Shy ME. Charcot-marie-tooth disease subtypes and genetic testing strategies. *Ann Neurol.* (2011) 69:22–33. 10.1002/ana.22166 21280073 PMC3058597

[4] Pareyson D. Charcot-marie-tooth disease and related neuropathies: molecular basis for distinction and diagnosis. *Muscle Nerve.* (1999) 22:1498–509. 10.1002/(sici)1097-4598(199911)22:113.0.co;2-9 10514227

[5] Reilly MM. Sorting out the inherited neuropathies. *Pract Neurol.* (2007) 7:93–105. 17430873

[6] Barreto LC Oliveira FS Nunes PS de França Costa IM Garcez CA Goes GM Epidemiologic study of Charcot-marie-tooth disease: a systematic review. *Neuroepidemiology.* (2016) 46:157–65. 10.1159/000443706 26849231

[7] Tai H Pan H Chen N Liang X Zhang Z. Investigation of clinical features on Charcot-marie-tooth disease type 2. *J Clin Neuro.* (2011) 24:327–30.

[8] Bienfait HM Baas F Koelman JH de Haan RJ van Engelen BG Gabreëls-Festen AA Phenotype of Charcot-marie-tooth disease type 2. *Neurology.* (2007) 68:1658–67. 10.1212/01.wnl.0000263479.97552.94 17502546

[9] Pogemiller K Garibay E Pierz K Acsadi G Õunpuu S. Comparison of gait patterns and functional measures between Charcot-marie-tooth disease type I and II in children to young adults. *Gait Posture.* (2020) 77:236–42. 10.1016/j.gaitpost.2020.01.027 32062403

[10] Lin KP Soong BW Yang CC Huang LW Chang MH Lee IH The mutational spectrum in a cohort of Charcot-marie-tooth disease type 2 among the Han Chinese in Taiwan. PLoS One. (2011) e29393:1–9. *Erratum PLoS One.* (2012) 7. 10.1371/journal.pone.0029393 22206013 PMC3242783

[11] Lee AJ Nam DE Choi YJ Nam SH Choi BO Chung KW. Alanyl-tRNA synthetase 1 (AARS1) gene mutation in a family with an intermediate Charcot-marie tooth neuropathy. *Genes Genomics.* (2020) 42:663–72. 10.1007/s13258-020-00933-9 32314272

[12] Tatsumi Y Matsumoto N Iibe N Watanabe N Torii T Sango K CMT type 2N disease-associated AARS mutant inhibits neurite growth that can be reversed by valproic acid. *Neurosci Res.* (2019) 139:69–78. 10.1016/j.neures.2018.09.016 30261202

[13] He W Zhang HM Chong YE Guo M Marshall AG Yang XL. Dispersed disease-causing neomorphic mutations on a single protein promote the same localized conformational opening. *Proc Natl Acad Sci USA.* (2011) 108:12307–12. 10.1073/pnas.1104293108 21737751 PMC3145702

[14] He W Bai G Zhou H Wei N White NM Lauer J CMT2D neuropathy is linked to the neomorphic binding activity of glycyl-tRNA synthetase. Nature. (2015) 526:710-4. *Erratum Nature.* (2016) 532:402. 10.1038/nature15510 26503042 PMC4754353

[15] Shy M Rebelo AP Feely SM Abreu LA Tao F Swenson A Mutations in BAG3 cause adult-onset Charcot-marie-tooth disease. *J Neurol Neurosurg Psychiatry.* (2018) 89:313–5. 10.1136/jnnp-2017-315929 28754666 PMC6152909

[16] Berghoff C Berghoff M Leal A Morera B Contreras C Barrantes R Late onset autosomal dominant Charcot-marie-tooth 2 neuropathy in a Costa Rican family. *Neurol Res.* (2009) 31:283–8. 10.1179/174313208X346080 18826755

[17] Auer-Grumbach M Strasser-Fuchs S Robl T Windpassinger C Wagner K. Late onset Charcot-marie-tooth 2 syndrome caused by two novel mutations in the MPZ gene. *Neurology.* (2003) 61:1435–7. 10.1212/01.wnl.0000094197.46109.75 14638973

[18] Zemmouri R Azzedine H Assami S Kitouni N Vallat JM Maisonobe T Charcot-marie-tooth 2-like presentation of an Algerian family with giant axonal neuropathy. *Neuromuscul Disord.* (2000) 10:592–8. 10.1016/s0960-8966(00)00141-3 11053687

[19] Wu DW Li Y Yin X Zhang B. A novel TFG c.793C>G mutation in a Chinese pedigree with Charcot-marie-tooth disease 2. *Brain Behav.* (2020) 10:e01724. 10.1002/brb3.1724 32666699 PMC7507360

[20] Padua L Pareyson D Aprile I Cavallaro T Quattrone DA Rizzuto N Natural history of Charcot-marie-tooth 2: 2-year follow-up of muscle strength, walking ability and quality of life. *Neurol Sci.* (2010) 31:175–8. 10.1007/s10072-009-0202-z 20016922

[21] Teunissen LL Notermans NC Franssen H Van Engelen BG Baas F Wokke JH. Disease course of Charcot-marie-tooth disease type 2: a 5-year follow-up study. *Arch Neurol.* (2003) 60:823–8. 10.1001/archneur.60.6.823 12810486

[22] Onder H Albayrak L Polat H. Frontal lobe ischemic stroke presenting with peripheral type facial palsy: a crucial diagnostic challenge in emergency practice. *Turk J Emerg Med.* (2017) 17:112–4. 10.1016/j.tjem.2017.04.001 28971160 PMC5608613

[23] Kuiyun M Xiaoxian S. *Analysis of Misdiagnosis of Nervous System Diseases.* Beijing: People’s Military Medical Press (2009). p. 556.

[24] Lin J Chen Y Wen H Yang Z Zeng J. Weakness of eye closure with central facial paralysis after unilateral hemispheric stroke predicts a worse outcome. *J Stroke Cerebrovasc Dis.* (2017) 26:834–41. 10.1016/j.jstrokecerebrovasdis.2016.10.029 27986397

[25] Wang Y Yin F. A review of X-linked Charcot-marie-tooth disease. *J Child Neurol.* (2016) 31:761–72. 26385972 10.1177/0883073815604227

[26] Santoro JD Chitnis T. Strokelike episodes in a patient with chronic gait abnormalities. *JAMA Neurol.* (2019) 76:621–2. 10.1001/jamaneurol.2019.0057 30801629

[27] Fugate JE Claassen DO Cloft HJ Kallmes DF Kozak OS Rabinstein AA. Posterior reversible encephalopathy syndrome: associated clinical and radiologic fifindings. *Mayo Clin Proc.* (2010) 85:427–32. 10.4065/mcp.2009.0590 20435835 PMC2861971

[28] Gewirtz AN Gao V Parauda SC Robbins MS. Posterior reversible encephalopathy syndrome. *Curr Pain Headache Rep.* (2021) 25:19. 10.1007/s11916-020-00932-1 33630183 PMC7905767

[29] Ohira J Mori N Kajikawa S Nakamura T Arisato T Takahashi M. Posterior reversible encephalopathy syndrome with extensive deep white matter lesions including the temporal pole. *Intern Med.* (2016) 55:3529–33. 10.2169/internalmedicine.55.7324 27904123 PMC5216157

[30] Li Y Castaldo J Bemporad J Yacoub HA. Reversible confluent deep white matter abnormalities: a new variant of posterior reversible encephalopathy syndrome. *Case Rep Neurol Med.* (2013) 2013:536978. 10.1155/2013/536978 24368950 PMC3866881

[31] Fourgeaud M Vidal T Schmitt C Blouin JM Ged C Richard E. Recurrent posterior reversible encephalopathy syndrome in a patient with acute intermittent porphyria. *Rev Neurol (Paris).* (2020) 176:118–20. 10.1016/j.neurol.2019.02.003 31153599

[32] Fugate JE Rabinstein AA. Posterior reversible encephalopathy syndrome: clinical and radiological manifestations, pathophysiology, and outstanding questions. *Lancet Neurol.* (2015) 14:914–25. 10.1016/S1474-4422(15)00111-8 26184985

[33] Sweany JM Bartynski WS Boardman JF. Recurrent posterior reversible encephalopathy syndrome: report of 3 cases–PRES can strike twice! *J Comput Assist Tomogr.* (2007) 31:148–56. 10.1097/01.rct.0000233127.21303.b9 17259848

[34] Lee VH Wijdicks EF Manno EM Rabinstein AA. Clinical spectrum of reversible posterior leukoencephalopathy syndrome. *Arch Neurol.* (2008) 65:205–10. 18268188 10.1001/archneurol.2007.46

[35] Girişgen I Tosun A Sönmez F Ozsunar Y. Recurrent and atypical posterior reversible encephalopathy syndrome in a child with peritoneal dialysis. *Turk J Pediatr.* (2010) 52:416–9. 21043390

[36] Donmez FY Agildere AM. Recurrent childhood PRES. *Neurol Sci.* (2015) 36:1603–9. 10.1007/s10072-015-2212-3 25894844

[37] Imaizumi N Takeuchi Y Hirano H Torii T Seki Y Morimoto T Data on the effects of Charcot-marie-tooth disease type 2N-associated AARS missense mutation (Arg329-to-his) on the cell biological properties. *Data Brief.* (2019) 25:104029. 10.1016/j.dib.2019.104029 31194127 PMC6554220

[38] Berciano J García A Gallardo E Peeters K Pelayo-Negro AL Álvarez-Paradelo S Intermediate Charcot-marie-tooth disease: an electrophysiological reappraisal and systematic review. *J Neurol.* (2017) 264:1655–77. 10.1007/s00415-017-8474-3 28364294

[39] Fischer M Schmutzhard E. Posterior reversible encephalopathy syndrome. *J Neurol.* (2017) 264:1608–16. 10.1007/s00415-016-8377-8 28054130 PMC5533845

[40] Servillo G Bifulco F De Robertis E Piazza O Striano P Tortora F Posterior reversible encephalopathy syndrome in intensive care medicine. *Intensive Care Med.* (2007) 33:230–6. 17119920 10.1007/s00134-006-0459-0

